# Endothelial Cells in Antibody-Mediated Rejection of Kidney Transplantation: Pathogenesis Mechanisms and Therapeutic Implications

**DOI:** 10.1155/2017/8746303

**Published:** 2017-02-01

**Authors:** Shuo Wang, Chao Zhang, Jina Wang, Cheng Yang, Ming Xu, Ruiming Rong, Tongyu Zhu, Dong Zhu

**Affiliations:** ^1^Department of Urology, Zhongshan Hospital, Fudan University, Shanghai, China; ^2^Shanghai Key Laboratory of Organ Transplantation, Shanghai, China; ^3^Shanghai Public Health Clinical Center, Fudan University, Shanghai, China

## Abstract

Antibody-mediated rejection (AMR) has been identified as a main obstacle for stable immune tolerance and long survival of kidney allografts. In spite of new insights into the underlying mechanisms of AMR, accurate diagnosis and efficient treatment are still challenges in clinical practice. Endothelium is the first barrier between recipients' immune systems and grafts in vascularized organ transplants. Considering that endothelial cells express a number of antigens that can be attacked by various allo- and autoantibodies, endothelial cells act as main targets for the recipients' humoral immune responses. Importantly, emerging evidence has shown that endothelial cells in transplants could also initiate protective mechanisms in response to immune injuries. A better understanding of the role of endothelial cells during the pathogenesis of AMR might provide novel therapeutic targets. In the present review, we summarize the antigens expressed by endothelial cells and also discuss the activation and accommodation of endothelial cells as well as their clinical implications. Collectively, the progress discussed in this review indicates endothelial cells as promising targets to improve current diagnosis and therapeutic regimens for AMR.

## 1. Introduction

Historically, cell-mediated rejection (CMR) was recognized as the predominant form of immune response in organ transplantation. However, progress in the last decade suggested that, besides CMR, antibody-mediated rejection (AMR) also significantly contributes to the rejection and pathogenesis of allografts [1, 2]. Despite the substantial advances in understanding the pathologic process of AMR, accurate diagnosis and efficient treatment are still challenges in clinic. This could be partly ascribed to our limited knowledge of the underlying mechanisms of AMR.

Vascular endothelium is the first barrier between recipients' immune system and allograft in solid organ transplantation. As endothelial cells express a number of antigens that can be targeted by various allo- and autoantibodies (Abs), endothelial cells play an important role in the pathogenesis of AMR [3–5]. Furthermore, increasing evidence has demonstrated that endothelial cells in allograft are not only passive participants, but also active regulators of pathophysiology in recipients [6]. Exploring the role of endothelial cells in AMR, therefore, will facilitate the improvement of current diagnosis and therapeutic regimens for AMR.

This review will summarize the cross talk between endothelial cells and antibodies in allograft rejection and its clinical relevance. We will also discuss the mechanism of activation and accommodation of endothelial cells and their clinical implications. Finally, we will put forward perspectives that could be a valuable subject of research in the future.

## 2. Endothelial Cells as Targets in Antibody-Mediated Rejection

### 2.1. Endothelial Antigens Targeted by Alloantibodies

#### 2.1.1. ABO Blood Group Antigens

As early as the 1900s, the ABO blood group system was discovered by Karl Landsteiner, who later won the Nobel Prize in Physiology or Medicine for this extraordinary contribution [7]. The ABO system is composed of genetically determined blood group antigens and corresponding antibodies (namely, isohaemagglutinins) in circulation [8]. Interestingly, these blood group antigens, including A, B, and H, are expressed not only on red blood cells, but also on other tissue cells, such as endothelial cells [9]. Anti-A/B antibodies are preformed natural antibodies, which are the main barriers for ABO-incompatible (ABOi) blood transfusions and organ transplantation. Early practice revealed that ABOi kidney transplantation without special treatment could result in unavoidable disastrous AMR [10, 11] (Table 1). In this respect, kidney transplantation that breaches the ABO system was considered an absolute contraindication for a long period of time. However, the organ-specific pattern of ABO antigens allows an exception for ABOi kidney transplantation. Individuals who are A2 subtypes express low levels of A antigens within kidneys [12]. Therefore, it is acceptable to perform incompatible transplant using kidneys from A2 donors even without adequate preconditioning [7]. With the improved understanding of the ABO-related AMR, ABO blood group compatibility has no longer been a prerequisite for kidney transplantation. Feasible desensitization regimens including anti-A/B antibody deletion and preemptive modulation of B-cell immunity have been developed and thus expand the donor pool significantly. More importantly, such transient treatment is able to induce long-term stable function of allografts even after the reappearance of anti-A/B antigens. This phenomenon is termed accommodation, which will be discussed later.

#### 2.1.2. Human Leukocyte Antigens (HLA)

HLA, also known as major histocompatibility complex, are genetically determined and highly heterogeneous proteins in human beings [13]. HLA is able to present antigens to T-cells and thereby regulate immune responses. There are 2 distinct classes of HLA that possess different functions. Class I HLA is expressed on all kinds of cells and exposes antigens within cells to CD8+ cytotoxic T-cells; class II HLA is expressed selectively on antigen presenting cells (APCs) as well as some special cell types, and they can present antigens to helper T-cells. Endothelial cells express both kinds of HLA.

HLA molecules themselves can also be recognized as antigens and induce allogeneic specific antibodies in the process of organ transplantation. The deleterious role of HLA antibodies has been studied extensively in the past decade. Preformed anti-HLA donor-specific antibodies (DSAs) due to pregnancies, blood transfusions, and organ transplantation contribute to higher risk for AMR and allograft failure [14]. Besides, the generation of de novo anti-HLA DSA is considered as a major risk factor for acute and chronic antibody-mediated rejection and graft loss, especially the complement fixing antibodies [15, 16].

#### 2.1.3. Major Histocompatibility Complex Class I Related Chain A Antigens (MICA)

MICA are highly polymorphic glycoproteins that are expressed on different types of cells including endothelial cells, and the expression of MICA can be induced upon stresses, which make them ideal targets in organ transplantation [17–19]. In 2002, Sumitran-Holgersson et al. identified preformed MICA antibodies as risk factor for graft loss [20]. Subsequent studies obtained similar results and showed that both preformed and de novo MICA antibodies could result in acute as well as chronic rejections [21–26].

It has been suggested that MICA-associated rejection is highly associated with C4d deposition [27, 28]. In addition, MICA antibodies were reported to cause cell death through complement-dependent cytotoxicity [29]. A very recent research showed that 23% of anti-MICA-positive sera from pretransplant patients could fix C1q and further confirmed that the deleterious effect of MICA antibodies relied on fixation and activation of the complement cascade [30]. All these data indicate an indispensable role of complement system in the pathogenesis of MICA antibodies.

Considering that MICA is not expressed on resting T- or B-cells, standard lymphocyte cross-matching fails to detect MICA antibodies [20, 31]. Mismatched MICA episodes in allografts are the main targets of MICA antibodies generated by recipients [32]. In this regard, it is of importance to perform MICA genotyping that is not available in the present clinical practice. Tonnerre et al. found that polyreactive anti-MICA sera bound preferentially to MICA*∗*008 (the most common allele of MICA) donor endothelial cells, indicating that MICA*∗*008(A5.1) molecules are the predominant determinants of MICA antibodies-related physiopathology [33]. A better understanding of MICA episodes in the background of kidney transplantation may provide feasible strategies for clinical monitoring and treatment.

### 2.2. Endothelial Targets of Autogenous Anti-Endothelial Cell Antibodies (AECAs)

#### 2.2.1. Angiotensin Type 1 Receptor (AT_1_R)

AT_1_R is a transmembrane G-protein coupled receptor that is expressed at the vascular endothelium [60]. Angiotensin II is the endogenous ligand for AT_1_R and exerts most of its effects through AT_1_R. Angiotensin II-AT_1_R signaling plays an important role in vasoconstriction, cell migration, protein synthesis, inflammation, and fibrosis in various physiologic and pathophysiologic context [61]. Recently, AT_1_R autoantibodies have drawn much attention due to their direct involvement in the pathogenesis of autoimmune diseases and solid organ allograft rejections [62, 63]. AT_1_R autoantibodies belong to IgG1 and IgG3 subclass and serve as AT_1_R agonists. Accordingly, malignant hypertension is recognized as one of the most prominent clinical symptoms in AT_1_R-associated disorders.

Dragun et al. first revealed the presence and pathogenic role of AT_1_R-Abs in a cohort of renal transplantation recipients with steroid-refractory vascular rejection and malignant hypertension in 2005 [34]. In this study, it is demonstrated that AT_1_R-Abs-positive and HLA-Abs-negative patients with vascular refection were at higher risk for allograft loss, in contrast to those with HLA-Abs and without AT_1_R-Abs.

Subsequently, other researches provided more evidence for the initial findings [35–47]. The largest retrospective study by now was conducted by Giral et al. in a cohort of 599 kidney transplant recipients [38]. In this study, the authors found that preformed AT_1_R-Abs were associated with a higher risk of acute rejection within the first 4 months after transplantation and graft failure after 3 years from transplantation. On the other hand, another research consisting of 351 patients demonstrated that, except for preexisting antibodies, de novo AT_1_R-Abs could also cause allograft failure [39].

#### 2.2.2. Endothelin-1 Type A Receptor (ET_A_R)

ET_A_R is a receptor for endothelin-1 and plays an important role in the regulation of blood pressure [64]. It is reported that anti-ET_A_R antibodies (ET_A_R-Abs) are strongly correlated with anti-AT_1_R antibodies in heart transplantation [65]. In renal transplantation, however, researches on ET_A_R-Abs are limited. Banasik et al. evaluated ET_A_R-Abs in a cohort of 116 kidney transplant recipients and found that ET_A_R-Abs existed in almost half of the recipients before transplantation and were related to reduced renal function and increased intimal arteritis after transplantation [48]. But there was no evidence that ET_A_R-Abs could deteriorate rejection rates. Another research from the same group demonstrated that ET_A_R-Abs were associated with higher risk for graft loss [41].

#### 2.2.3. Vimentin

Vimentin is an intermediate filament protein existing within cells of mesenchymal origin, including endothelial cells. Upon the settings of endothelial injuries, vimentin is exposed to the immune system and thereby results in the production of autoantibodies against vimentin [49, 66]. There have been studies demonstrating the presence of anti-vimentin antibodies (AVA) in kidney transplantation [49, 50]. Besarani et al. further correlated AVA with interstitial fibrosis and tubular atrophy of kidney allografts in a retrospective study including 70 recipients [50]. An experimental research of murine cardiac transplant demonstrated the colocalization of AVA and C3d on endothelium of allografts, indicating a vital role of complement in the AVA-mediated injuries [67]. The destructive effect of AVA, however, seemed to be dependent on alloimmune responses, for vascular lesions were not observed in syngeneic hearts.

#### 2.2.4. Perlecan

Perlecan is an important component of vascular basement membrane that contains 3 laminin-like globular (LG) domains in its C-terminal [68]. LG3 can be cleaved from perlecan and elicits the production of autoantibodies during endothelial injuries [69, 70]. It has been shown that increased serum level of LG3 itself was highly associated with acute vascular rejection [51]. Moreover, another report from the same group revealed elevated anti-LG3 IgG titers before and after transplantation in kidney recipients with acute vascular rejection rather than those with tubule-interstitial rejection or normal graft function [52]. It should be noted that patients who were concomitantly positive for both pretransplantation DSAs and posttransplantation LG3 antibodies had inferior graft survival, indicating the synergy effect between DSAs and LG3 antibodies.

#### 2.2.5. Endoglin, FLT3 Ligand, EDIL3, and ICAM4

Recently, four novel targets on endothelial cells for AECAs were identified using high-density protein arrays: endoglin, Fms-like tyrosine kinase 3 ligand (FLT3 ligand), EGF-like repeats and discoidin I-like domain 3 (EDIL3), and intercellular adhesion molecule 4 (ICAM4) [53]. Enzyme linked immunosorbent assay was performed to detect these AECAs in a validation cohort consisting of 151 renal recipients. Result showed that these four AECAs were obviously related to HLA-DSAs and AMR.

## 3. Endothelial Cells Act as Participants in AMR: Activation versus Accommodation

Vascular endothelium is the main interface for the direct contact between recipients' immune system and allografts in kidney transplants as well as other solid organ transplantation procedures. Considering abundance of antigens expressed by endothelial cells as discussed previously, vascular endothelial cells serve as a preferential target for host immune response. However, endothelial cells are not only “passive victims” in the settings of transplantation, but also “active participants” in this pathophysiologic process. Notably, accumulating evidence indicated endothelial cells as a potential promoter for immune tolerance. Therefore, it is of vital importance to understand the role and mechanism of endothelial cells in modulating immune responses and allograft pathogenesis.

### 3.1. Activation of Endothelial System: Cross Talk between Endothelial and Immune Cells

Activation of endothelial cells refers to the proinflammatory transition under certain microenvironment based on resting condition [71]. Classically, endothelial activation can be divided into 2 distinguished types. Type I activation is induced by histamine or thrombin and type II activation is initiated in response to cytokines such as TNF-*α* or IL-1. Type I activation acts as a quick fashion independently of de novo gene transcription. In contrast, type II activation relies on gene expression and thereby exhibits a slower process. Activation of endothelial cells could result in various pathophysiologic effects, of which the most important one in the context of allograft rejection is the recruitment and priming of circulating leukocytes. Expression of adhesion molecules and chemokines contributes to this process.

It should be noted that endothelial cells are semiprofessional APCs and are able to activate T-cells, including CD8+ and CD4+ T-cells [72]. In this context, it is of interest to consider whether endothelial cells could exert a direct effect on B-cells and humoral immunity. Given the indispensable role of helper T-cells in the generation of antibodies, endothelial cells are proposed to influence antibody production indirectly via presenting self-antigens to helper T-cells.

Interestingly, a recent research found that endothelial cells could also recruit regulatory T-cells (Tregs) [73]. Recognition of self-antigens of endothelial cells plays a key role in the trafficking of Tregs into target organs. And this recruitment effect of endothelial cells is dependent on IFN-*γ*-associated microenvironment. Such accumulated Tregs contribute to peripheral immune tolerance. However, how this process could be related to organ transplantation remains unclear and requires further investigation.

Moreover, endothelial cells may also participate in graft nephropathy through regulating thrombosis. Normally, resting endothelial cells could maintain blood fluidity and regulate blood flow. Upon activation, however, they upregulate procoagulant molecules expression and subsequently promote thrombi formation [6].

### 3.2. Accommodation: Resistance to Allograft Rejection

Nowadays, ABOi transplantation has become a routine option for kidney recipient candidates. This breakthrough, to a great extent, is ascribed to the long-term acceptance of allografts after transient treatment of desensitization. Even though the titers of anti-A/B antibodies increased again after a period of time, ABOi transplant recipient could avoid the assumed higher risk for AMR and keep allograft function stable, which is termed “accommodation” [74]. Current perspectives recognize accommodation as the self-protection and resistance of endothelial cells against AMR. The scientific community has paid much attention to the exploration of the underlying mechanisms of accommodation and determining whether this protective effect could be augmented in clinic practice.

Various mechanisms of endothelial cell-mediated graft protection have been reported by a number of studies (Table 2). In 1997, Bach et al. reported that heart xenografts could acquire accommodation by upregulation of a number of antiapoptotic and anti-inflammatory genes including A20, Bcl-2, Bcl-xl, and hemeoxygenase-1 in endothelial cells [54]. Accordingly, similar mechanisms have been confirmed in renal grafts in the subsequent studies. Salama et al. examined endothelial behavior during accommodation in renal recipients [55]. Immunohistochemistry of the graft biopsies demonstrated increased expression of antiapoptotic protein Bcl-xl in glomerular and peritubular capillary endothelial cells. The authors further performed in vitro experiments to confirm that endothelial cells with upregulated Bcl-xl were rendered resistant to complement-dependent cytotoxicity. Chen et al. reported that antiapoptotic proteins and complement regulatory proteins such as Bcl-2, CD59, CD46, and clusterin might contribute to allografts' accommodation [56]. Interestingly, Iwasaki et al. compared molecular signaling of accommodation under different conditions in vitro [57]. They found that accommodation for anti-A/B antibodies relied on unregulated complement regulatory proteins CD55 and CD59 induced by suppressed ERK1/2 pathway, whereas in the background of anti-HLA antibodies activated PI3K/AKT pathway of endothelial cells led to expression of cytoprotective molecules such as hemeoxygenase-1 and ferritin H. These results indicated that, specifically, induction of anticomplement or antiapoptosis molecules on endothelial cells might be a promising strategy to improve antirejection regimens in clinic. However, the mechanism in depth and feasible treatment modality needs further investigation.

Another explanation for accommodation is the ABO blood group changes on endothelial cells. A study by Tanabe et al. showed time-dependent downregulation of donor's blood-type antigen on the graft endothelium, which might contribute to the long-term accommodation after ABOi kidney transplantation [58]. Besides, the same group confirmed detectable antigenic chimerism on the graft endothelium in another research [59]. The establishment of antigenic chimerism is still not fully understood and warrants further exploration.

Taken together, although substantial breakthroughs have been made in researches of endothelial accommodation, it is still not feasible to develop endothelial cell-targeted therapeutic strategies currently. Investigations therefore are urgently needed in the future.

## 4. Endothelial Cell-Related Diagnostic Biomarkers in AMR

AMR is recognized as the major obstacle for long survival of kidney grafts. Efficient treatment for AMR relies on accurate diagnosis. The diagnosis of AMR, however, is sophisticated due to the paucity of characteristic hallmarks under heterogeneous conditions. Recently, several literatures exhibited that some molecular markers of endothelial activation were highly connected to AMR and were able to serve as diagnostic indicators.

In 2009, Sis et al. screened 119 endothelial-associated transcripts in 173 renal grafts to determine their possible role in diagnosis of AMR [75]. They found that increased expression of kidney endothelial transcript successfully predicts active antibody-mediated allograft damage and poor graft outcome. The result was confirmed in an independent validation cohort containing 82 kidneys. Predictive endothelial markers were further explored subsequently. Most recently, a study from Xu-Dubois et al. discovered that endothelial-to-mesenchymal transition (EMT) is of vital importance in the process of AMR, and 3 EMT markers, that is, fascin1, vimentin, and heat shock protein 47, are sensitive and reliable markers for diagnosis for AMR [76]. Taken together, exploration for predictive markers in endothelial cells might provide alternatives for accurate diagnosis for AMR.

## 5. Final Remarks

Thanks to the progress in organ preservation and immunosuppressive regimens, 1-year survival of kidney allografts has reached 95%. However, the improvements in long-term graft survival are limited and remain unsatisfactory. AMR is recognized as one of the leading causes of graft loss. In this regard, understanding the underlying mechanisms of AMR will facilitate better therapeutic strategies.

Due to the abundance of surface and inside antigens, vascular endothelial cells act as preferential targets for both allo- and autoantibodies. More importantly, endothelial cells in allograft are not only passive participants, but also active regulators in the process of AMR. Upon injuries or inflammation, endothelial cells can increase the expression of allo- and autoantigens, as well as adhesion molecules and chemokines, and thereby recruit and activate circulating leukocytes. On the other hand, endothelial cells are able to initiate self-protection pathways under similar conditions. The balance between their proinflammatory capacities and accommodation statement might decide the final fate of the allograft. Therefore, it is of great value to explore how to modulate this balance favorably towards reducing immunogenicity and increasing graft acceptance.

Taken together, endothelial cells are indispensable participants in the pathophysiology of AMR, and therapeutics targeted at endothelial cells hold great promise to improve the current immunosuppressive regimens, which warrant urgent researches in the near future.

## Figures and Tables

**Table 1:** Endothelial antigens in antibody-mediated immune responses.

Types of Abs	Endothelial antigens	Time course of Ab formation	Hyperacute rejection	Acute rejection	Long-term graft injury	Reference
Alloantibodies	ABO	Preformed & de novo	Yes	Yes	Yes	[10, 11]
	HLA	Preformed & de novo	Yes	Yes	Yes	[14–16]
	MICA	Preformed & de novo	Yes	Yes	Yes	[20–33]

Autoantibodies	AT_1_R	Preformed or de novo	No	Yes	Yes	[34–47]
	ET_A_R	Preformed or de novo	No	No	Yes	[41, 48]
	Vimentin	De novo	No	No	Yes	[49, 50]
	Perlecan	Preformed or de novo	No	Yes	Yes	[51, 52]
	Endoglin	Preformed	No	Yes	N/A	[53]
	FLT3 ligand	Preformed	No	Yes	N/A	[53]
	EDIL3	Preformed	No	Yes	N/A	[53]
	ICAM4	Preformed	No	Yes	N/A	[53]

**Table 2:** The proposed mechanisms of endothelial cell-mediated accommodation in ABOi transplantation.

Study	Design	Key findings	Reference
*Alleviation of apoptosis and complement*			
Bach et al.	Hamster to rat heart xenografts	Heart xenografts could acquire accommodation by upregulation of a number of antiapoptotic and anti-inflammatory genes including A20, Bcl-2, Bcl-xl, and hemeoxygenase-1 in endothelial cells.	[54]
Salama et al.	Human renal transplantation with HLA antibodies	Immunohistochemistry of the graft biopsies demonstrated increased expression of antiapoptotic protein Bcl-xl in glomerular and peritubular capillary endothelial cells. In vitro experiments confirmed that endothelial cells with upregulated Bcl-xl were rendered resistant to complement-dependent cytotoxicity.	[55]
Chen et al.	Renal transplantation in skin-presensitized nonhuman primates	Antiapoptotic proteins and complement regulatory proteins such as Bcl-2, CD59, CD46, and clusterin might contribute to allografts' accommodation.	[56]
Iwasaki et al.	In vitro study of the effects of anti-HLA and anti-A/B antibody binding on complement-mediated cytotoxicity and signal transduction	Accommodation for anti-A/B antibodies relied on unregulated complement regulatory proteins CD55 and CD59 induced by suppressed ERK1/2 pathway, whereas in the background of anti-HLA antibodies activated PI3K/AKT pathway of endothelial cells led to expression of cytoprotective molecules such as hemeoxygenase-1 and ferritin H.	[57]

*Blood group alteration or chimerism*			
Tanabe et al.	ABOi renal transplant recipients	Time-dependent downregulation of donor's blood-type antigen on the graft endothelium was observed, which might contribute to the long-term accommodation after ABOi kidney transplantation.	[58]
Tanabe et al.	ABOi renal transplant recipients	Detectable antigenic chimerism on the graft endothelium was confirmed.	[59]

## Abbreviations

Ab: Antibody
ABOi: ABO-incompatible
AECA: Autogenous anti-endothelial cell antibody
AMR: Antibody-mediated rejection
APC: Antigen presenting cell
AT_1_R: Angiotensin type 1 receptor
AVA: Anti-vimentin antibodies
CMR: Cell-mediated rejection
DSA: Donor-specific antibody
EDIL3: EGF-like repeats and discoidin I-like domain 3
EMT: Endothelial-to-mesenchymal transition
ET_A_R: Endothelin-1 type A receptor
HLA: Human leukocyte antigen
FLT3: Fms-like tyrosine kinase 3
ICAM4: Intercellular adhesion molecule 4
LG: Laminin-like globular domain
MICA: Major histocompatibility complex class I related chain A antigen
Treg: Regulatory T-cell.
