# Corrigendum to “Endothelial Cells in Antibody-Mediated Rejection of Kidney Transplantation: Pathogenesis Mechanisms and Therapeutic Implications”

**DOI:** 10.1155/2019/9691679

**Published:** 2019-03-10

**Authors:** Shuo Wang, Chao Zhang, Jina Wang, Cheng Yang, Ming Xu, Ruiming Rong, Tongyu Zhu, Dong Zhu

**Affiliations:** ^1^Department of Urology, Zhongshan Hospital, Fudan University, Shanghai, China; ^2^Shanghai Key Laboratory of Organ Transplantation, Shanghai, China; ^3^Shanghai Public Health Clinical Center, Fudan University, Shanghai, China

In the article titled “Endothelial Cells in Antibody-Mediated Rejection of Kidney Transplantation: Pathogenesis Mechanisms and Therapeutic Implications,” [1] there was an error in a funding number, where “Grants 8150056 to Dong Zhu” should be corrected to “Grants 81500569 to Dong Zhu.” The corrected Acknowledgments section is as follows:

This review was supported by the National Natural Science Foundation of China (Grants 81500569 to Dong Zhu and 81570674 to Tongyu Zhu).
